# The relationship between myodural bridges, hyperplasia of the suboccipital musculature, and intracranial pressure

**DOI:** 10.1371/journal.pone.0273193

**Published:** 2022-09-02

**Authors:** Chan Li, Chen Yue, Zhao-Chang Liu, Jin Gong, Xiao-Song Wei, Heng Yang, Campbell Gilmore, Sheng-Bo Yu, Gary D. Hack, Hong-Jin Sui

**Affiliations:** 1 Department of Anatomy, Dalian Medical University, Dalian, China; 2 Department of Gynecology and Obstetrics, The Second Hospital of Dalian Medical University, Dalian, China; 3 Medical School, St. George’s University of London, London, United Kingdom; 4 Department of Advanced Oral Sciences and Therapeutics, University of Maryland, Baltimore, Maryland, United States of America; Max Delbruck Centrum fur Molekulare Medizin Berlin Buch, GERMANY

## Abstract

During mammalian evolution, the Myodural Bridges (MDB) have been shown to be highly conserved anatomical structures. However, the putative physiological function of these structures remains unclear. The MDB functionally connects the suboccipital musculature to the cervical spinal dura mater, while passing through the posterior atlanto-occipital and atlanto-axial interspaces. MDB transmits the tensile forces generated by the suboccipital muscles to the cervical dura mater. Moreover, head movements have been shown to be an important contributor to human CSF circulation. In the present study, a 16-week administration of a Myostatin-specific inhibitor, ACE-031, was injected into the suboccipital musculature of rats to establish an experimental animal model of hyperplasia of the suboccipital musculature. Using an optic fiber pressure measurement instrument, the present authors observed a significant increase in intracranial pressure (ICP) while utilizing the hyperplasia model. In contrast, surgically severing the MDB connections resulted in a significant decrease in intracranial pressure. Thus, these results indicated that muscular activation of the MDB may affect CSF circulation, suggesting a potential functional role of the MDB, and providing a new research perspective on CSF dynamics.

## Introduction

The suboccipital region is one of the most complex anatomical regions in the human body, and is composed of the occiput (C0), the atlas (C1), the axis (C2) and the associated suboccipital muscles [1]. The suboccipital region plays a significant role in movements of the head and neck as well as the transmission of physiological signals traveling between the brain and spinal cord [2].

In 1995, a bridge-like anatomical structure was described passing through the atlanto-occipital interspace in humans, connecting the cervical dura mater to the rectus capitis posterior minor (RCPmi) muscle and was named the “Myodural Bridge” (MDB) [3]. During the following two decades, other MBD-like structures were observed connecting the cervical dura mater to other suboccipital muscles, including the rectus capitis posterior major (RCPma) and the oblique capitis inferior (OCI) muscles [4–8]. The MDB structures have now been identified passing through both the atlanto-occipital and the atlanto-axial interspaces in humans. Moreover, the MDB has been shown to be an evolutionary conserved structure in other species as well, including terrestrial mammals such as dogs, cats, rats, macaques, and rabbits; marine mammals such as the finless porpoise, and the sperm whale; and reptiles such as the crocodile [9–12].

However, to date, the putative physiological function of the MDB in mammals remains unclear, and there is no experimental evidence to indicate its physiological significance. Up until now the putative function of the MDB has been primarily based on its shape, location, and histological properties. The MDB has been suggested to be primarily fixing device for the cervical dura mater, preventing the dura mater from folding inward while the suboccipital muscles are overextended; and pulling the cervical dura mater to maintain the subarachnoid space open [13–20].

Cerebrospinal fluid (CSF) circulation is a much more complex system than previously believed existing within the central nervous system (CNS) of humans. Unlike the blood circulation system, there are no smooth muscles within the human CNS, and thereby the dynamic mechanism of CSF circulation remains a puzzle to researchers. Currently, it is believed that the main drivers of CSF circulation involve arterial pulsations, breathing, body position, and intracranial blood circulation [21–23]. A publication in 2014 revealed that instead of a pure directional flow, the CSF can flow non-directionally from high pressure areas to low pressure areas, as the pressure changes in various cavities (including the ventricles, central spinal cord, and subarachnoid space, etc.) within the CNS [24].

Considering the MDB, the suboccipital muscles, and the cervical dura mater as a unit, whenever the suboccipital muscles are activated, the MDB transmits the tensional forces, generated by the suboccipital musculature, to the cervical dura mater. The MDB then pulls the dura mater, changing the pressure within the subarachnoid space, leading to pressure changes in various cavities in the CNS, and ultimately affecting the dynamics of CSF circulation. Additionally, changes of intracranial pressure (ICP) have been shown to produce headaches [25]. Moreover, a number of studies by our research group have evidenced that: a) most patients with clinically unexplained headaches have hyperplasia of their RCPmi muscles [19]; b) human head movements lead to instantaneous changes of the velocity and quantity of CSF flow [20].

Myostatin (also called GDF-8) is a protein secreted by myocytes. This protein inhibits the proliferation and differentiation of myocytes, plays a role in balancing muscle size, and participates in regulating muscular functions [26]. Individuals lacking myostatin exhibit significantly increased muscle mass. Furthermore, studies have shown that artificially inhibiting the ions produced by myostatin within muscles (such as the use of myostatin inhibitors), leads to a stronger physique in experimental animals, as well as significantly increased muscular strength. Moreover, myostatin knockout mice also evidenced a stronger physique and greater muscle tone [27]. Furthermore, the overexpression of genes related to muscle atrophy, such as MuRF-1 and Atrogin-1, can be used as markers of muscle hyperplasia and muscle function [28].

Ace-031 is a myostatin-specific inhibitor approved for clinical use in 2016. Ace-031 competitively binds to myostatin proteins, antagonizes the normal myostatin receptor ACTVIIb, and blocks the downstream signaling pathway of myostatin, resulting in a physiological dysfunction of myostatin proteins [29]. For the present study involving myostatin, we delivered Ace-031 to the rectus capitis dorsalis major (RCDma) and rectus capitis dorsalis minor (RCDmi) muscles of the experimental animals via local injection, thus developing a long-term RCDma and RCDmi-hyperplasia animal model. In contrast, we used an electric knife to sever the RCDma and RCDmi muscles in another group of rats that served as the negative control. In recent years, a novel approach of ICP measurement using optic-fiber pressure transducer had been introduced [30]. By analyzing the ICP changes of each group of animal, we were able to determine whether the MDB participates in the dynamic regulation of CSF circulation, and thus to reveal the putative physiological function of the MDB. Collectively, these results further supported our theory concerning CSF dynamics and provided a theoretical basis for the clinical treatment of diseases related to abnormal and unexplained intracranial pressure.

## Materials and methods

### Ethics statement

The present study was performed in strict accordance with the recommendations presented in the guide for the Care and Use of Laboratory Animals of the National Institutes of Health. The protocol was approved by the Committee on the Ethics of Animal Experiments of the Dalian Medical University. All surgery was performed under Avertin anesthesia, and every effort was made to minimize suffering.

### Animals

The experimental rats utilized in the present study were all males and were descended from different female rats. The rats were housed until 12-week old and then used for the subsequent experiments. All rats were housed on a 12 hr light cycle and fed standard rodent diet (Medicience Ltd. Jiangsu, China) and water ad libitum. The present study was carried out in strict accordance with the recommendations stated in the guide for the Care and Use of Laboratory Animals from the National Institutes of Health. The protocol was approved by the Committee on the Ethics of Animal Experiments at the Dalian Medical University. All surgery was performed under anesthesia, and every effort was made to minimize suffering. For the present study, three research groups of experimental animals were established. A total of thirty-six animals were used in the present study and twelve animals for each group.

### Surgery and local injection of Ace-031

**Fig 1:**
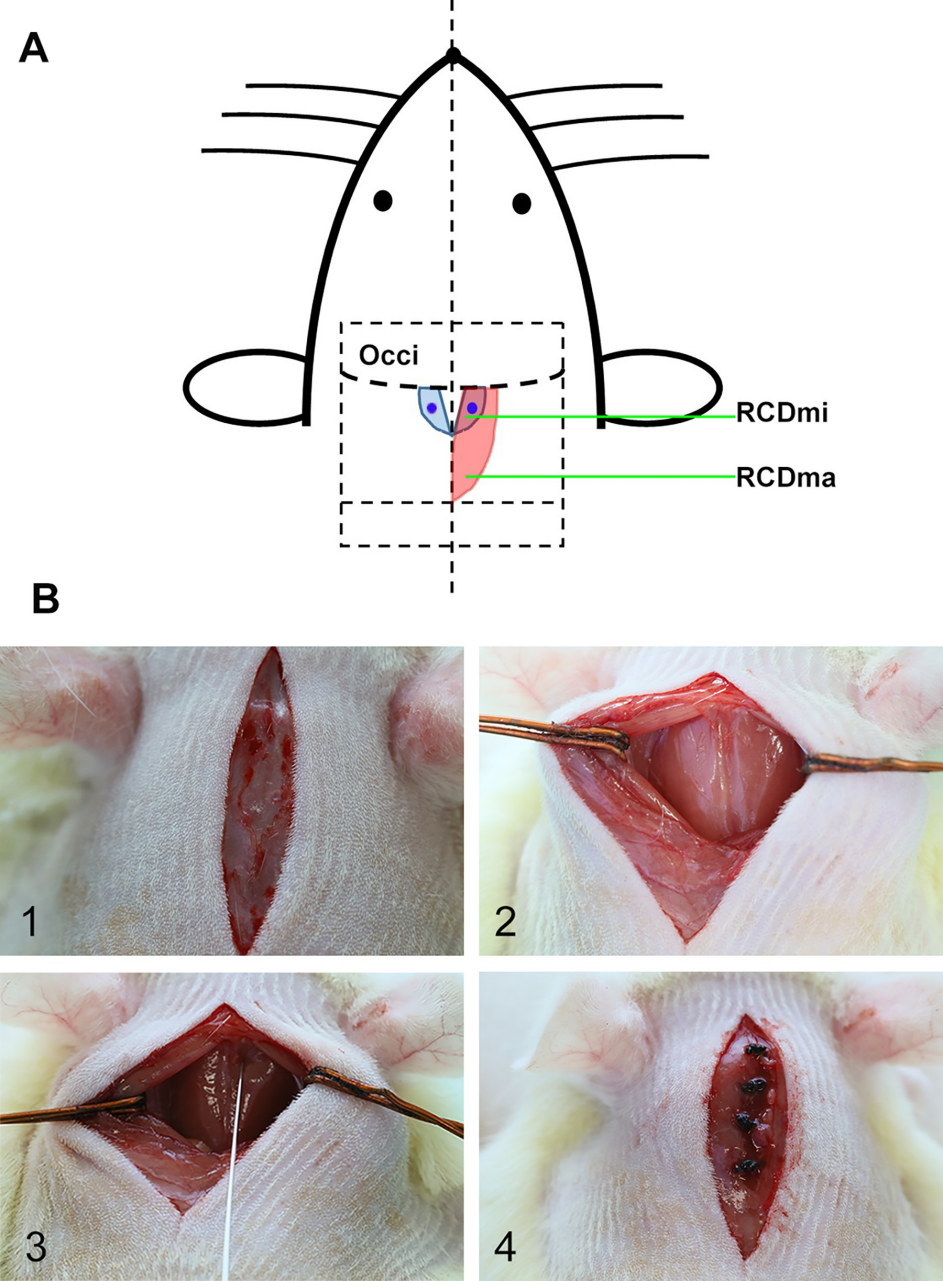
Surgical preparation and Ace-031 injections. A. modeling diagram of local injection; B. operation method: 1. prepare the skin of the surgical region and make a sagittal incision on the skin along the midline, 2. make a sagittal incision on the superficial layer of muscles to expose the RCDma muscles, 3. locate the injection site and inject, 4. suture the superficial layer of muslces and skin respectively. RCDma: rectus capitis dorsalis major; RCDmi: rectus capitis dorsalis minor; blue circle: injection site.

All rats receiving local injections were fasted overnight for the subsequent surgical procedures. For their anesthesia, a 2.5% Avertin solution was applied (i.p.). A sagittal incision was made on the dorsal aspect of the rat’s neck (Fig 1B1); the skin and the superficial layer of muscles were then lifted aside to expose the RCDma muscles (Fig 1B2). For the Myostatin (GDF-8) local blocked group of rats (GDF-8LB, n = 12), the injection site was located 2mm below the inferior margin of the occiput, and 5mm to the left and right of the midline (Fig 1A). 25μl micro injection syringes with a caliber of 0.22mm (32G), was used for all the injections (Hamilton, NY, USA). The angle of the injections was made perpendicular to the inferior surface of the occiput. The needle was withdrawn approximately 5mm until it was pulled away from the RCDma, after contacting the posterior surface of the occiput. The injection was administered at a constant speed during this entire process (Fig 1B3), the superficial layer of muscles and the skin were then returned to its original position and sutured respectively at last (Fig 1B4). According to the body weight of each rat, 5mg/kg of Ace-031 (AbMole Bioscience, Shanghai, China) was injected into the RCDmi muscles, with the maximal volume not exceeding 20μl. For the control group of rats (GDF-8WT, n = 12), the local injection with same volume of saline was performed following the same injection method. For the occipital muscles severed group of rats (SEV, n = 12), a same sagittal incision was made on skin and the superficial layer of muscles to expose the suboccipital muscles. Next the RCDma as well as RCDmi muscles were severed near the lower edge of the occiput, and the severed ends of the RCDma and RCDmi muscles were then cauterized with an electric thermal cautery device (Geiger Medical Technologies, Inc. IA, USA) to prevent the muscles from reconnecting to the occiput. Serving as the internal control for the GDF-8LB group, a local injection of Ace-031, with a similar final concentration of 5mg/kg, was given to the right gastrocnemius muscle of the rats. 200,000 units of penicillin was injected i.p. to prevent post-operative infection after surgery. The local injection of Ace-031 was repeated weekly to achieve a constant blockage of myostatin (GDF-8) expression. The injection of saline was repeated weekly as well.

### Intracranial pressure measurement

The intracranial pressure (ICP) for each experimental group was measured after 16-week consecutive local injections. A fiber optic pressure transducer (i-evo 669, FISO technology Inc., Canada) was utilized to measure the intracranial pressures (ICP). Rats receiving the 2.5% Avertin (i.p.) anesthesia were fixed on a stereotaxic apparatus (Model 789311N, Benchmark, USA) in the prone position. Next a 1.5cm midline skin incision was made on scalp. (Sterile instruments and gloves were used.). Blunt dissection of the soft tissues and surrounding muscles was performed to locate the Lambda and Bregma rat skull landmarks. The skin and connective tissues were then retracted. Using a dental drill with a 1mm burr, a 2mm wide perforation was made into the right parietal bone. The perforation was made to be located 2mm lateral and 2mm posterior to the Bregma landmark to avoid the superior sagittal sinus and to ensure that the ICP sensor was placed over the ischemic territory. A 5cm long transparent plastic tube, with an outer diameter equal to that of the bony perforation, was inserted into the skull opening. A pipette was utilized to mix and apply a clear dental cement to the base of the surgical boney perforation and around the inserted plastic tube. The ICP probe was then inserted 5cm into the tube so that the tip of the probe was level with the end of the tube. This careful placement of the probe assured that the tip of the probe did not touch or perforate the cranial dura mater. Checked the ICP value on the screen, the base line should be at zero, the waveform should be nothing more than a horizontal line. Continue to insert the probe 0.01mm at a time. Before the tip touched the dura, the waveform on the sensor screen was a horizontal line, once the tip made contact with the dura, a slight wave could be seen (very slight not yet a strong and typical wave, any of the amplitudes should be shorter than 0.1mmHg), at this time point we pressed the calibration button, the baseline was set to 0mmHg. After that, the tip of the probe was inserted another 0.01mm to achieve a steady ICP tracing reflecting ventilation and blood pressure pulse waves. For airtight, all of the gaps between the bony perforation and the tube were then sealed with a colored dental polymer. This colored cement was allowed to set for 5 minutes before any readings were made.

During the measurement procedure, for each rat toe pinch test was performed every 2–3 minutes. The anesthesia ICP value at the 20th minute after Avertin injection was collected as the internal control, at this point of time none of the rats showed a toe pinch response; all of the rats started to show toe pinch response after the 25th minute, and the measurement was continued after this time point; all of rats exhibited spontaneous head movements after the 35th minute, and the last stable ICP value right before unable to continue the measurement due to behavioral interference of awakening (such as a sudden rising waveform due to spontaneous head movements and so), was collected as the awaken ICP value. The awaken ICP values were normalized against values of internal control for final statistical analysis.

The skin was returned to its original position and sutured with 12-gauge thread, 200,000 units of penicillin was injected i.p. to prevent post-operative infection. At least 8 rats of each group were engaged in the ICP measurements.

### Western blot

Utilizing the western blot (protein immunoblot) analytical technique, the protein concentrations were quantified using the BCA method (Micro BCA protein Assay Reagent kit; Thermo Fisher Scientific Inc. Waltham, MA, USA). 20μg of protein were applied to each lane. To determine the expression levels of myostatin, equal amounts of protein from the RCDma, the RCDmi and gastrocnemius muscles obtained from the testing group (GDF-8LB, n = 4) as well as the control group (GDF-8WT, n = 4), were subjected to western blot. After 16-week consecutive local injections, the rats were sacrificed after ICP measurements. Next the RCDma, the RCDmi, and the gastrocnemius muscles were removed and frozen in liquid nitrogen. Equal amounts of protein, extracted from the frozen tissues, were subjected to western blot. For use in all the western blots, the anti-myostatin (GDF-8) was produced in goats (AF788, R&D systems, MN, USA) and the anti-α-tublin was produced in rabbits (KG22771, KeyGEN BioTECH Co., Beijing, China). Additionally, Horseradish peroxidase-conjugated anti-Goat IgG (GE Healthcare) and horseradish peroxidase-conjugated anti-Rabbit IgG (GE Healthcare) were used as secondary antibodies. These antibodies were diluted in TBS with Tween-20 (TBST) in 5% skim milk. ECL prime (GE Healthcare) was then used to detect the bands, according to the commercial protocol instructions.

### Real-Time PCR

For real-time RT-PCR quantification of the muscle mRNA levels of atrogin-1 and MuRF-1, the rats were fasted for 36 hours. Next, mRNA was extracted from the muscles using the Trizol method (Life Technologies. Inc). The following primers were used for RT-PCR: atrogin-1 forward 5’- GCA GAG AGT CGG CAA GTC-3’, reverse 5’- CAG GTC GGT GAT CGT GAG-3’; MuRF-1 forward 5’-GCC ATC CTG GAC GAG AAG AAG-3’, reverse 5’-AGC GGC TTG GCA CTC AAG-3’. For all RT-PCR quantification, GAPDH was used as the internal control, GAPDH forward 5’-GTG GAC CTC ATG GCC TAC AT-3’, reverse 5’-TGT GAG GGA GAT GCT CAG TG-3’. RT-PCR was carried out using the SYBR green method (Takara Bio Inc. Shiga, Japan). Atrogin-1 and MuRF-1 mRNA levels were normalized against GAPDH mRNA. Four rats of each group (GDF-8WT, GDF-8LB) were engaged in this test after ICP measurements.

### Histological and microscopy

Skeletal muscles were isolated from both the fasted testing group (GDF-8LB, n = 8) and the control group (GDF-8WT, n = 8) of rats and immediately fixed with a formalin solution (Sigma). After regular paraffin embedding, serial 8- mm sections were then made. The hematoxylin and eosin (HE) stained specimens were photographed utilizing a Nikon NIS image system (Nikon Eclipse 80i, Nikon, Tokyo, Japan). Myocyte cross-sectional areas were quantified using Image-J software (N.I.H.). At least 100 myocytes were measure per animal.

### Statistics

Statistical analysis was carried out using Prism 7 (Graphpad Software) and Microsoft Excel Statistics Toolkit (Microsoft). Student’s t-tests were applied. Results shown are mean±SD, a p-value of <0.05 was considered to be statistically significant.

## Results

### The blockage from myostatin resulted in an increased myocyte size in the RCDma and RCDmi muscles

**Fig 2:**
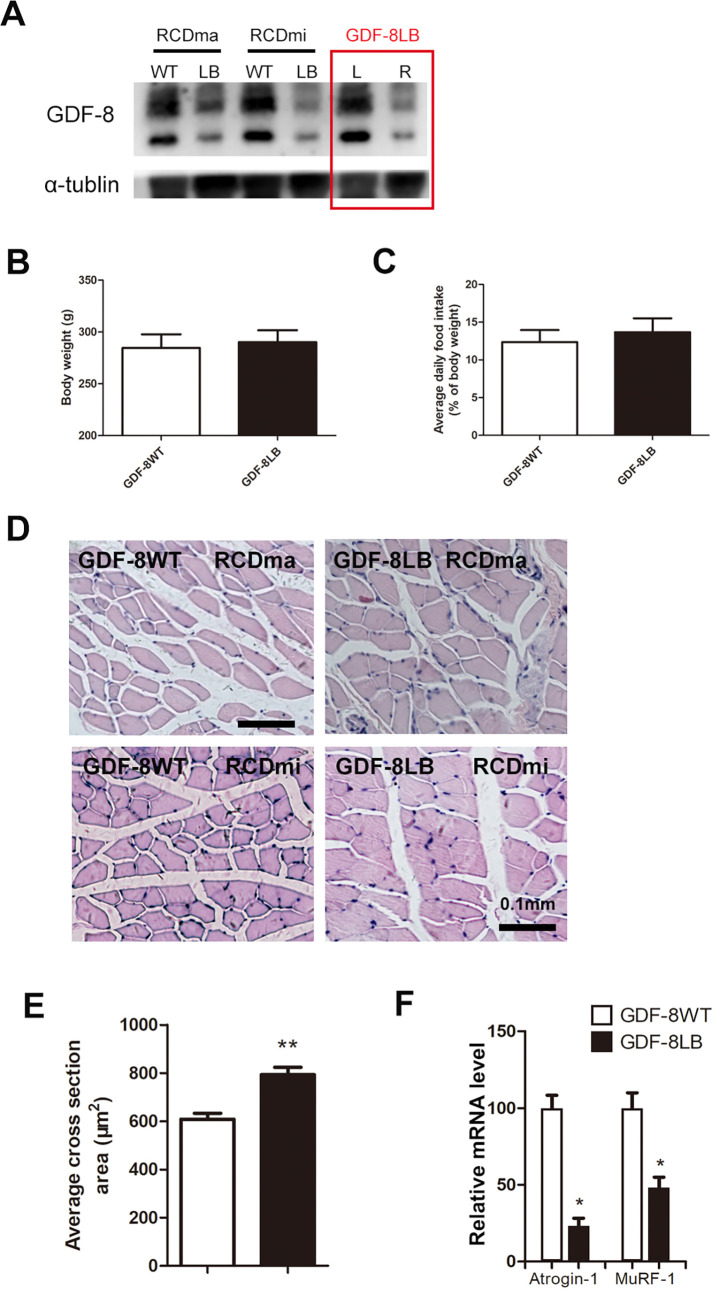
The blockage from myostatin resulted in an increased myocyte size in the RCDma and RCDmi muscles. A, expression level of myostatin (GDF-8) in the RCDma and RCDmi muscles in GDF-8WT and GDF-8LB rats, and the left and right gastrocnemius muscles in GDF-8LB rats, after consecutive local injection of Ace-031 for 16 weeks; B. comparison of body weight; C, comparison of average daily food intake; D. representative cross-sections of RCDma and RCDmi muscles with H&E stain; E. average myocyte cross sections of the RCDma and RCDmi muscles in GDF-8WT and GDF-8LB rats; F. relative mRNA expression levels of Atrogin-1and MuRF-1 after 16-week consecutive local injection of Ace-031. (❊, P<0.05, student t-test, A and F, n = 4; B and C, n = 12; D and E, n = 8).

A suboccipital muscle hyperplasia model, employing a 16-week trial, involving injections of a Myostatin specific inhibitor (Ace-031), was performed on both the RCDma and the RCDmi muscles. The injection was repeated once a week during the total 16-week time span. Injections of Ace-031 were also delivered to the right gastrocnemius muscle which served as the internal control. With the rats receiving the local muscle blockage of Myostatin (GDF-8LB), we measured the levels of Myostatin (GDF-8) utilizing western blot analysis. We observed the inhibitory effect of the injections on the GDF-8 gene in the three injected muscles. The expression levels of the GDF-8 gene in the left gastrocnemius muscles remained unchanged (Fig 2A).

Myostatin (GDF-8) signaling has been implicated in muscle growth and fiber size. Rats with Myostatin blockage have evidenced significant muscle hypertrophy [27]. For the present study, we investigated whether the rats receiving the local injections of Ace-031 would experience muscle hypertrophy. The body weight of both the GDF-8LB and the GDF-8 wild type (GDF-8WT) rats were determined and related to their average daily food intake. Although the rats in the GDF-8LB group evidenced a decline in their average body weight and an increased average daily food intake, the differences did not reach statistical significance (Fig 2B & 2C). In addition, we examined fiber size in the RCDma and RCDmi muscles from both the GDF-8LB and the GDF-8WT rats. Neither of these two groups showed any histological muscle fiber abnormalities. However, the GDF-8LB rats appeared to have wider cross-sectional areas of their muscle fibers when compared to the GDF8-WT rats (Fig 2D). Moreover, according to the morphometric analysis, the RCDma and RCDmi muscle fibers from the GDF-8LB rats showed a 30% increase of in their mean cross-sectional areas (Fig 2E). The E3 ubiquitin ligases, Atrogin-1 and MuRF-1, are important regulators of ubiquitin-mediated protein degradation in skeletal muscles. Prolonged fasting has been reported to trigger the expression of these two genes and induce protein degradation [31, 32]. Real-time PCR results of the mRNA levels of these two genes expressed in the RCDma and RCDmi muscles from the GDF-8LB rats were markedly lower than the levels observed in the control rats (GDF-8WT) at fast sate (Fig 2F), Thus, suggesting that the increased myocyte size and increased muscle weight is related to the observed muscle protein degradation. These results further suggest that local blockage of GDF-8 leads to increased muscle weight, with this muscle weight increase being due to the increased size of the muscle fibers, which were observed in both the RCDma and RCDmi muscles.

### Local blockage of myostatin resulted in increased muscle mass and enhanced muscle strength in the right gastrocnemius muscles

**Fig 3:**
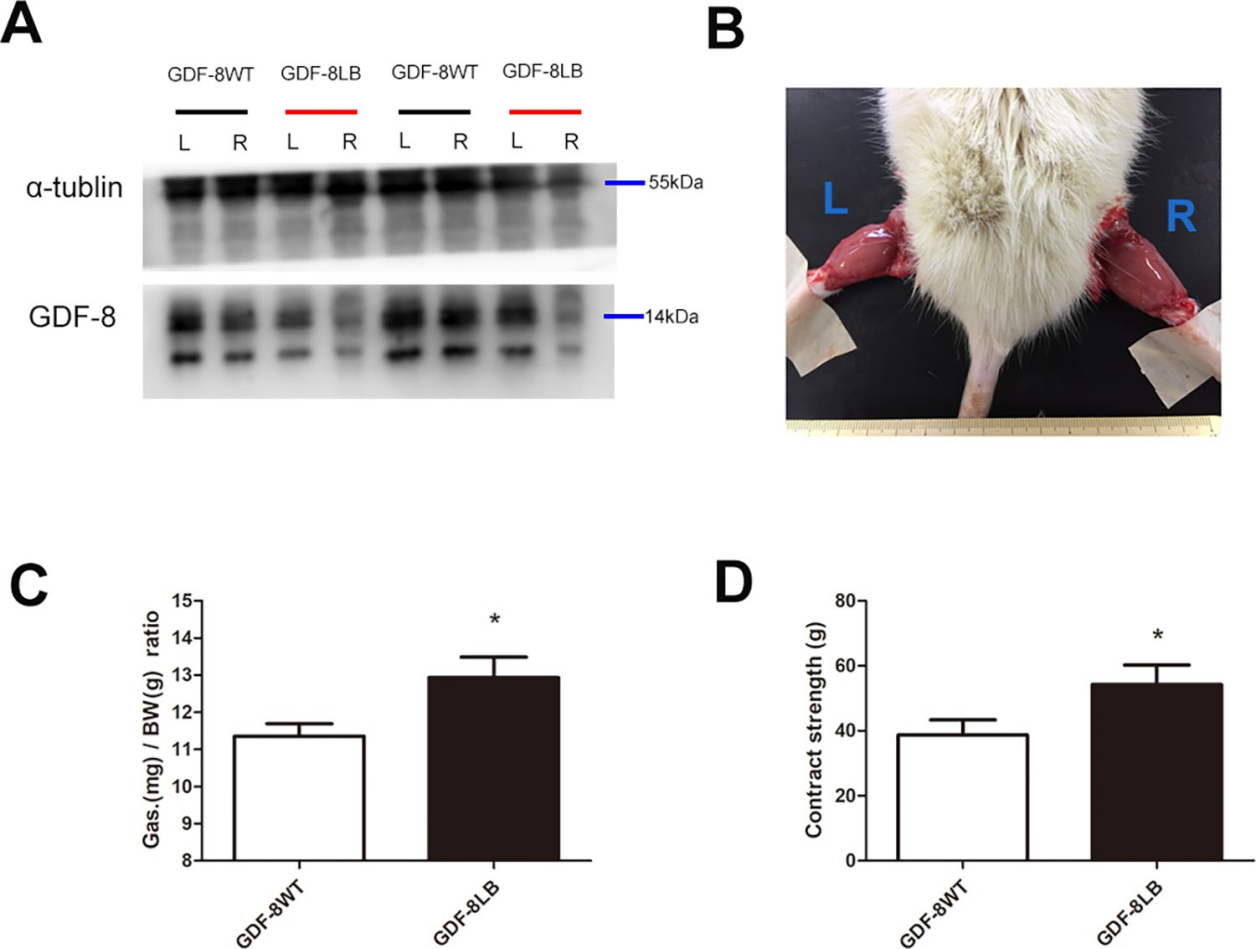
Local blockage of myostatin resulted in increased muscle mass and enhanced muscle strength in the right gastrocnemius muscles. A. expression level of myostatin (GDF-8) in the left and right gastrocnemius muscles in either GDF-8WT and GDF-8LB rats, after consecutive local injection of Ace-031 for 16 weeks; B, comparison of left and right gastrocnemius muscles of GDF-8LB rats after consecutive local injection of Ace-031 for 16 weeks; C. comparison of the gastrocnemius/body weight ratio; D. contraction force of right gastrocnemius muscle. (*, P<0.05; student t-test, n = 4).

Generally, skeletal muscle hypertrophy leads to enhanced muscle strength. Although the RCDma and RCDmi muscles both showed hyperplasia in the GDF-8LB rats, these two pairs of muscles are actually too small to accurately measure their contraction strengths via the electrical stimulation method. Therefore, after 16-week of consecutive local injection of Ace-031, we checked the weights as well as the tensile strength of the right gastrocnemius muscles in the GDF-8LB rats. To evaluate the effect of local inhibition, the expression levels of GDF-8 were checked and the results demonstrated a successful local blockage in the right gastrocnemius muscle of the GDF-8LB rats (Fig 3A). Moreover, as predicted, we found that the right gastrocnemius muscles of the GDF-8LB rats showed an increase in muscle mass (Fig 3B). The right gastrocnemius muscles of the GDF-8LB rats were observed to be approximately 20% heavier than the left gastrocnemius muscles when normalized to body weight (Fig 3C). In addition, we tested the contraction strength of the right gastrocnemius muscles of the GDF-8LB and the control rats (GDF-8WT). We found that the average contraction strength of the right gastrocnemius muscles showed a 25% increase compared to the control rats when applying a 20MHZ electrical stimulus (Fig 3D). Although we did not directly test the contraction strength of the RCDma and RCDmi muscles in the GDF-8LB rats, it is probable that the local blockage of Myostatin (GDF-8) would have also led to decreased muscle strength in these two pairs of suboccipital muscles, which serve as the primary origins of the MDB.

### Hyperplasia of the suboccipital muscles significantly increased intracranial pressure

**Fig 4:**
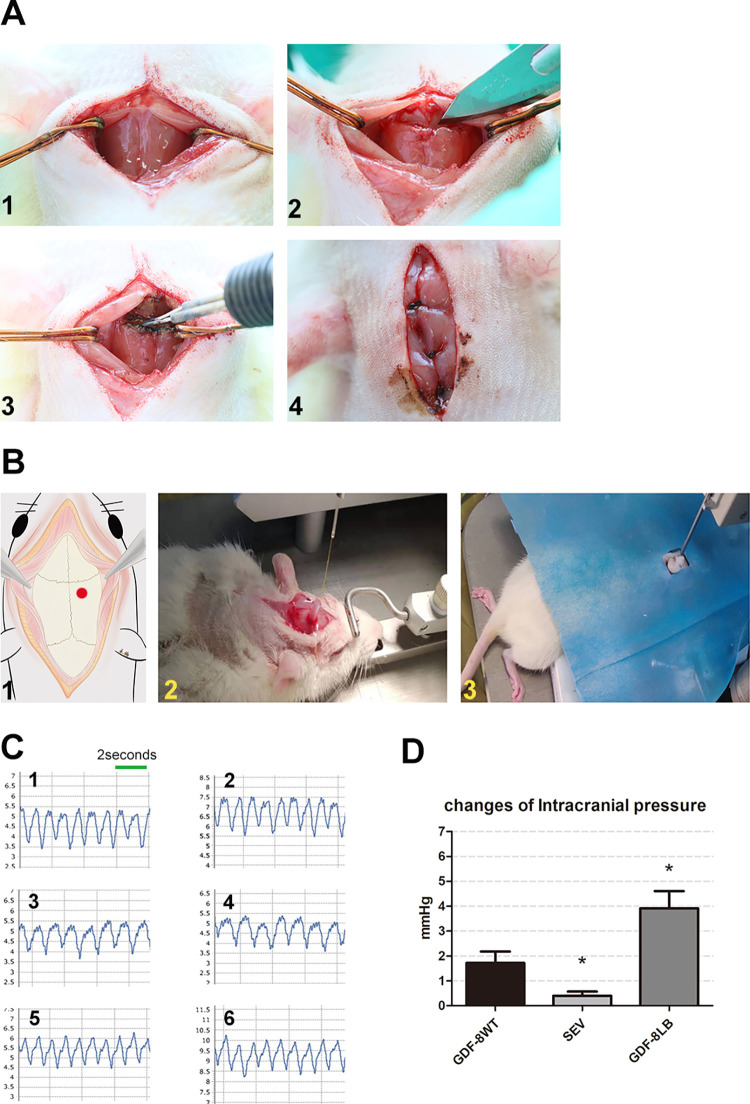
Hyperplasia of the suboccipital muscles significantly increased intracranial pressure. A. 1, a sagittal incision was made on skin and the superficial layer of muscles to expose the RCDma muscles; 2, the RCDma as well as RCDmi muscles were severed near the posterior edge of the occiput; 3, the severed ends of the RCDma and RCDmi muscles were then cauterized; 4, the superficial layer of muscles and skin were sutured at last. B. 1, perforation site is 2mm lateral and 2mm posterior to the bregma; 2, a transparent plastic tube was inserted into the skull opening and a clear dental cement was applied to seal the gap between the boney perforation and the inserted plastic tube; 3, the ICP probe was fixed for measurement and all the gaps were sealed gain with another colored dental polymer. C. 1, representative anesthesia ICP level of saline injection group (GDF8-WT); 2, representative awaken ICP value of saline injection group (GDF-8WT); 3, representative anesthesia ICP level of RCDma and RCDmi muscles severed group (SEV); 4, representative awaken ICP level of RCDma and RCDmi muscles severed group (SEV); 5, representative anesthesia ICP level of Ace-031 local injection group (GDF8-LB); 6, representative awaken ICP level of Ace-031 local injection group (GDF-8LB); D, overall changes of ICP levels (awaken ICP levels normalized against anesthesia ICP levels). *, P<0.05, student t-test, n≥8.

As the MDB connects the suboccipital muscles, including the RCDma and RCDmi, to the cervical dura mater, it was important to also evaluate the intracranial pressure (ICP) of the rats in the hyperplasia animal model group. For this reason, we used a group of rats to serve as the negative control by severing and disconnecting the RCDma and RCDmi muscles from their origin on the posterior aspect of the occiput. Following the operation method of local injection, a sagittal incision was made at first, the RCDma as well as RCDmi muscles were severed around the lower edge of the occiput, the cut ends were then cauterized to prevent the reconnection to the occiput (Fig 4A). The awaken intracranial pressure (ICP) of the rats with local injection of Ace-031 (GDF-8LB), the control group rats with local injection of saline (GDF-8WT), and the rats with the severed RCDma and RCDmi muscles (SEV), were all measured and normalized against ICP values under anesthesia condition (Fig 4B and 4C). The ICP values of the rats in the SEV group showed a moderate attenuation but with statistical significance; in contrast, the ICP values of the hyperplasia rats (GDF-8LB) increased significantly. These results indicate that the hyperplasia as well as the hypermyotonia of the RCDma and RCDmi muscles can lead to a significant increase in ICP (Fig 4D).

## Discussion

Since the initial report of the existence of the MDB in humans in 1995 [3], multiple studies have been carried out verifying the universal existence of this anatomical structure. The MDB has now been identified not only in humans, but also in a wide range of other animals, including dogs, rats, rabbits, macaques, and guinea pigs within the mammalian species, siamensis crocodiles and red-eared sliders (*Trachemys scripta elegan*) within the reptilian species, and pigeons within the avian species [33]. However, the physiological function of the MDB has previously been inferred primarily based on its anatomical features, including its shape, location, neighboring structures, and its histological properties. It is suggested that 1) the MDB actively and passively pulls the cervical dura mater to ensure that the dura mater does not improperly fold when the suboccipital muscles are overextended, 2) the MDB acts as a proprioceptor, limiting and preventing excessive flexion and extension of the head, and 3) the MDB helps to maintain the proper position of the spinal cord by pulling on the dura mater [13–20]. However, the precise physiological function of the MDB remains unclear.

For a universally present anatomic structure that has now been identified in multiple animal species, it most likely represents a physiologically significant structure, due to its survival throughout the natural selection of evolution. In 2017, our research group found that head rotations modulate human CSF circulation. We demonstrated that head rotations immediately change the direction as well as the velocity of CSF flow in humans [20]. Thus, the suboccipital region in humans must play a significant role in CSF circulation. As the MDB connects the suboccipital muscles to the cervical dura mater, we hypothesized that the MDB system modulates CSF circulation.

Various types of animal models, relating to myostatin, have been developed to study muscle atrophy and dystrophy, which includes Mdx mice and other whole-body transgenic mice. In these rodents, the single myostatin allele has been knocked out at birth. Other researchers developed another type of myostatin-disabled rat by administering intravascular injections of a specific myostatin inhibitor, Ja-16. This type of animal model becomes more manageable because of the ability to control the injection rate. Nonetheless, these animal models represent whole-body myostatin disabled animals, in which blockage of the myostatin protein is non-selective and affects the entire body of the animals. After considering these animal models, and our own research design, we performed injections of a new myostatin inhibitor, Ace-031, on the RCDma and RCDmi muscles, which serve as the primary origin of the MDB.

There are various viewpoints regarding the relationship between the MDB and CSF circulation. One study in 1997 inferred that the MDB functioned to maintain the flow of CSF within the subarachnoid cavity and the cisterna magna (cerebellomedullary cisterna) during head movements. These investigators proposed that the RCDmi muscle prevents the “infolding” of the dura mater during head movements, which may inhibit CSF flow [34]. Another recent publication suggested that the MDB is related to the maintenance of CSF outflow from the cisterna magna by maintaining the integrity of the subarachnoid space [35]. In 2017, our research team investigated CSF flow before and after head rotations, using a phase-contrast cine magnetic resonance imaging method. We demonstrated that head rotations affect the mean velocity, the flow rate, and the flow direction of CSF within the occipito-cervical junction [20]. Experimental results of another recent publication revealed that the body movements of a snake (viper boa) produced greater CSF pulses than those generated by the snakes cardiac and/or ventilatory cycles [36]. In summary, these studies enhanced our confidence regarding our own hypothesis that the MDB may play a crucial role in the modulation of CSF circulation. How can such a small anatomical structure, the MDB, be associated with significant pathophysiological functions? Hofmann’s ligaments are restricted to the lower lumbar spine and the upper sacral canal, and were initially considered to function simply as a fixing device of the lumbar dura mater, without having any functional significance [37]. Most researchers inferred that Hofmann’s ligaments act to maintain the dura mater against the vertebrae, as the spine lengthens [38]. It is now believed that Hofmann’s ligaments have a functional significance. Hofmann’s ligaments connect the lumbar dura mater to the nerve roots and the posterior longitudinal ligament. Whenever the intervertebral discs are injured, Hofmann’s ligaments transduce excessive tensional forces to the nerve roots, which leads to an increase in tension on the nerve roots, producing pain [39].

**Fig 5:**
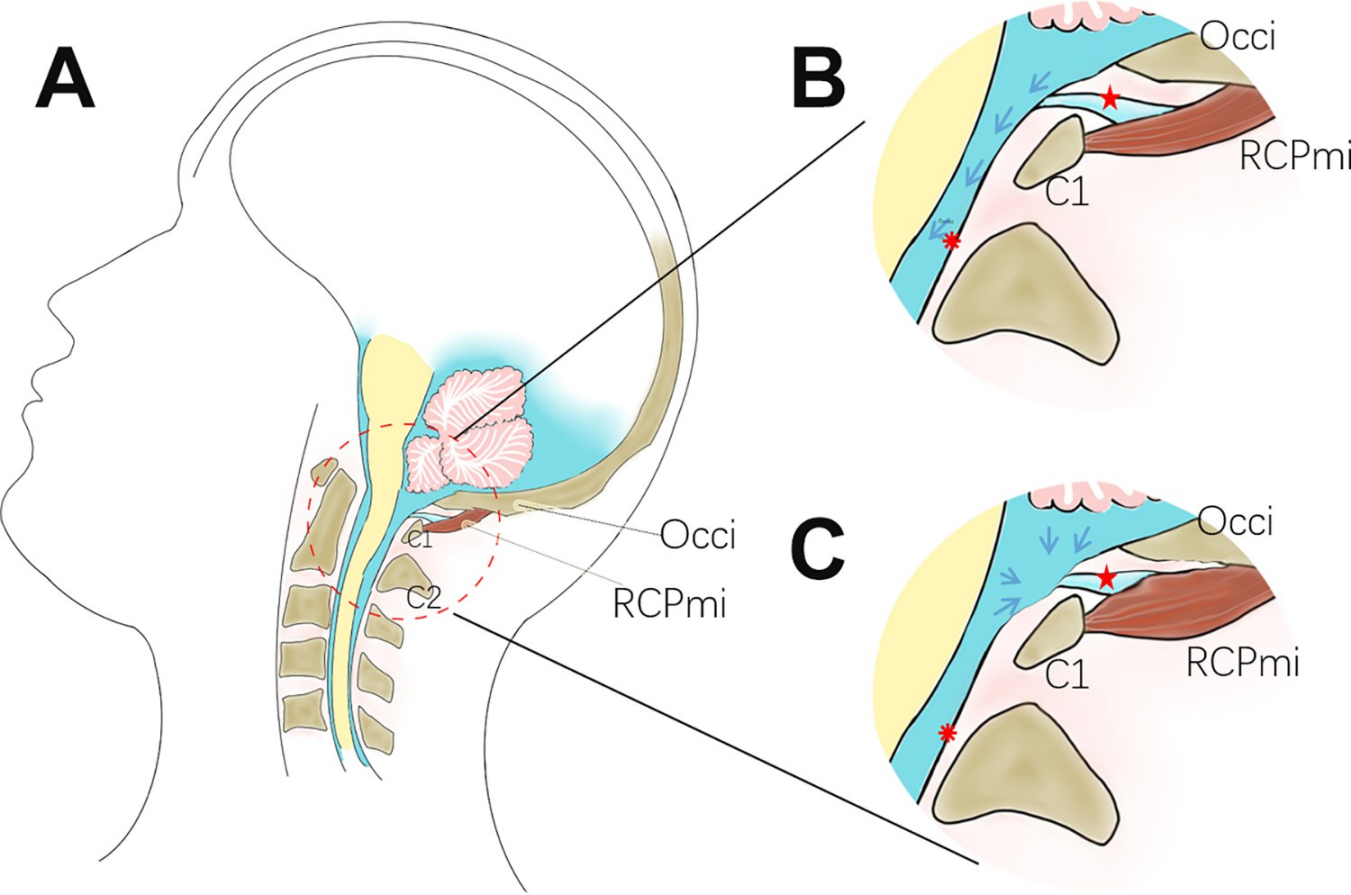
Hyperplasia of suboccipital muscles affects the CSF movement via the MDB. A. the CSF circulation at normal physiological state; B. enlarged view of the red circled region of A., the blue arrows indicate normal dynamics of CSF circulation. C. At the state of hyperplasia of the RCPmi, the MDB is passively stretched to change the volume of the subarachnoid space at the cervical level. C1: atlas; C2: axis; Occi: Occiput; RCPmi: rectus capitis posterior minor; red asterisk: cervical dura mater; red star: MDB (myodural bridge).

Moreover, regarding CSF circulation, it is widely accepted that pulsations produced by the arteries serve as a major power source to pump the CSF. Our study in 2018 revealed that the sperm whale has an extremely strong MDB when compared to terrestrial mammals [11]. The diving heart rate of the sperm whale can decrease down to 10 beats/min [40]. According to recent research, the blue whale can reduce its heart rate down to 3 beats/min while diving to a maximum depth [41]. This significantly decreased heart rate can critically slow down the flow rate of CSF circulation, creating a potentially fatal condition for these marine animals. Therefore, the MDB of these whales may contribute to transferring the tensile forces, generated by their suboccipital muscles, to the cervical dura mater. Thus, continuously altering the volume of CSF within the subarachnoid space, acting as a unique mechanism to circulate the CSF. Therefore, the MDB may play a key role in maintaining CSF circulation in these animals. Furthermore, there is not only a uni-directional flow of CSF circulation, as the CSF can flow from high-pressure regions to low-pressure regions. As the MDB connects the RCDma and RCDmi muscles to the cervical dura mater, it may transfer tensional forces to the cervical dura mater, changing the volume of the subarachnoid space at the cervical level (Fig 5). In the present study, we used rats and not marine mammals, and yet we still observed a statistically significant increase in the rats ICP. Additionally, in the negative control group with the severed suboccipital muscles, we observed a significant decrease in the intracranial pressure. These results supported our hypothesis that the MDB plays a significant role in modulating CSF circulation.

Clinically, many types of headaches are associated with an abnormal increase in ICP. In the present study, we injected Ace-031 into the gastrocnemius muscle in the right leg of each rat in the testing group serving as the internal control. The results revealed that the muscles were not only enlarged, but their contraction force was enhanced as well. Hypertrophy of the RCDma and RCDmi muscles resulted in a significant increase in tensional forces that could be transmitted to the cervical spinal dura via the MDB. This changed the volume of the local subarachnoid space and increased the intracranial pressure.

In the present study, we reported that the rats in the hypertrophy model displayed a significant increase in ICP, and in the severed muscle group the rats had a significant decrease in ICP. These results support our hypothesis that the MDB is a significant contributor to CSF circulation. This finding could be a key point in revealing the pathogenesis of some unexplained chronic headaches. Regarding this animal model, the behavioristics, as well as the velocity and quantity of CSF circulation should be further studied to further elucidate CSF dynamics. Moreover, this animal model may help to clarify the precise physiological significance of the MDB, and provide a unique therapeutic prospective for physicians relating to unexplained long-term headaches.

## Supporting information

S1 Raw images Original gel information for WesternBlot. A, the equivalent copy of Fig 2A and 2B, the equivalent copy of Fig 3A and 3C, original gel information of Fig 2A and 2D, original gel information of Fig 3A.
(TIF)
