# Supplementary figures and images for: The relationship between myodural bridges, hyperplasia of the suboccipital musculature, and intracranial pressure
Source: PLoS One. 2022 Sep 2;17(9):e0273193. doi: 10.1371/journal.pone.0273193 (PMC9439232; doi:10.1371/journal.pone.0273193)

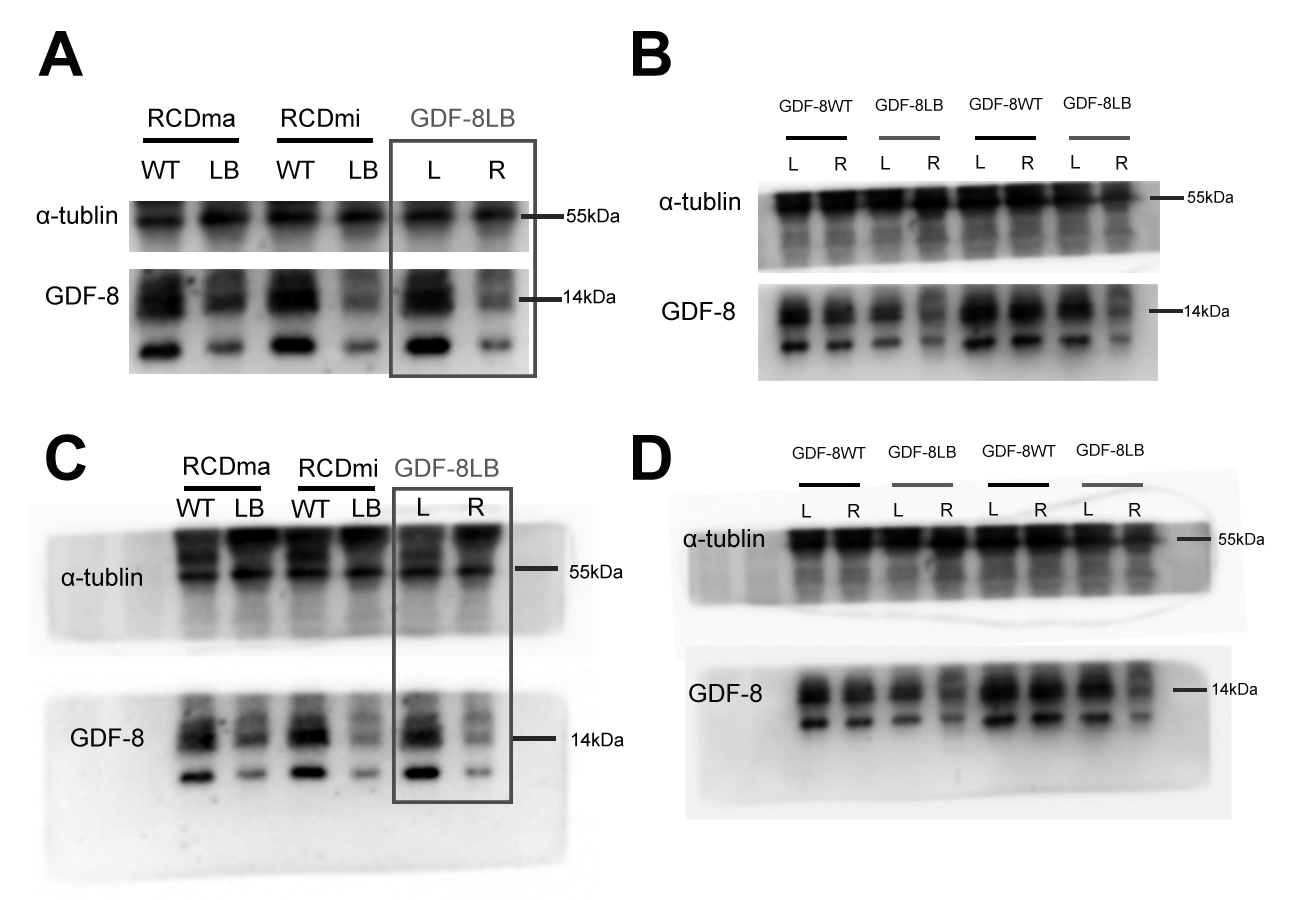

Supplement: S1 Raw images — A, the equivalent copy of Fig 2A and 2B, the equivalent copy of Fig 3A and 3C, original gel information of Fig 2A and 2D, original gel information of Fig 3A. (TIF) [file pone.0273193.s001.tif]
